# Synthesis of *N*-(6-Arylbenzo[d]thiazole-2-acetamide Derivatives and Their Biological Activities: An Experimental and Computational Approach

**DOI:** 10.3390/molecules21030266

**Published:** 2016-02-25

**Authors:** Yasmeen Gull, Nasir Rasool, Mnaza Noreen, Ataf Ali Altaf, Syed Ghulam Musharraf, Muhammad Zubair, Faiz-Ul-Hassan Nasim, Asma Yaqoob, Vincenzo DeFeo, Muhammad Zia-Ul-Haq

**Affiliations:** 1 Department of Chemistry, Faculty of Science and Technology, Government College University Faisalabad, Faisalabad 38000, Pakistan; yasmeenchem1@yahoo.com (Y.G.); mnazanoreen@yahoo.com (M.N.); nr_308@hotmail.com (M.Z.); 2 Department of Chemistry, Faculty of Science, University of Sargodhah, Bhakkar Campus, Bhakkar 30000, Pakistan; 3 Department of Chemistry, Faculty of Science, University of Gujrat, Hafiz Hayat Campus, Gujrat 50700, Pakistan; atafali_altaf@yahoo.com; 4 International Center for Chemical and Biological Sciences, Hussain Ebrahim Jamal Research Institute of Chemistry, University of Karachi, Karachi 75270, Pakistan; musharraf1977@yahoo.com; 5 Department of Chemistry, Faculty of Science, Islamia University of Bahawalpur, Bahawalpur 63000, Pakistan; faiznasim@hotmail.com (F.-H.N.); asmayaqoobctn@gmail.com (A.Y.); 6 Department of Pharmaceutical and Biomedical Sciences, University of Salerno, Via Ponte don Melillo, Fisciano (Salerno) I-84084, Italy; 7 Offices of Research, Innovation and Commercialization, Lahore College for Women University, Lahore 54600, Pakistan; ahirzia@gmail.com

**Keywords:** Suzuki cross coupling, Pd(0) catalyst, benzothiazole, nitric oxide scavenging activity, antiurease activity, haemolytic activity

## Abstract

A new series of *N*-(6-arylbenzo[d]thiazol-2-yl)acetamides were synthesized by C-C coupling methodology in the presence of Pd(0) using various aryl boronic pinacol ester/acids. The newly synthesized compounds were evaluated for various biological activities like antioxidant, haemolytic, antibacterial and urease inhibition. In bioassays these compounds were found to have moderate to good activities. Among the tested biological activities screened these compounds displayed the most significant activity for urease inhibition. In urease inhibition, all compounds were found more active than the standard used. The compound *N*-(6-(*p*-tolyl)benzo[d]thiazol-2-yl)acetamide was found to be the most active. To understand this urease inhibition, molecular docking studies were performed. The *in silico* studies showed that these acetamide derivatives bind to the non-metallic active site of the urease enzyme. Structure-activity studies revealed that H-bonding of compounds with the enzyme is important for its inhibition.

## 1. Introduction

Benzothiazoles consist of a benzene ring fused with a thiazole ring. Various benzothiazole derivatives serve as drugs, dyes and industrial chemicals [1,2,3]. Benzothiazoleand its derivatives such as esters have also been reported as active against Gram-positive and Gram-negative bacteria such as *Staphylococcus epidermidis*, *Escherichia coli*, *Enterobacter* and yeast (*Candida albicans*) [4]. Benzothiazole derivatives have also found to possess anticancer, antifungal and antibacterial activities [5,6]. 2-Aminobenzothiazole and a number of other aminobenzothiazole derivatives have been reported as muscle relaxants [7,8]. A literature survey performed for the current study revealed that 6-substituted-2-aminobenzothiazole derivatives such as 6-methyl, 6-methoxy, 6-ethoxy and 6-isopropoxy show antibacterial, anti-inflammatory and analgesic properties [9]. Various other derivatives are found to be cytotoxic against various tumors [10,11].

Suzuki cross coupling reactions are remarkable methods for C-C bond formation, utilized for the synthesis of agrochemicals, advanced materials and pharmaceuticals at both the industrial and laboratory scale [12,13,14,15,16,17].

The purpose of this study was to synthesize novel *N*-(6-arylbenzo[d]thiazol-2-yl)acetamides employing the Pd(0)-catalyzed Suzuki cross coupling methodology. This article describes our optimized experiments for the synthesis of *N*-protected 6-bromobenzothiazoles. The biological activities of these newly synthesized molecules were studied with the intention to explore their potential as future drugs. We investigated urease inhibition, nitric oxide scavenging, haemolytic and antibacterial activities. Molecular docking studies were performed to determine how they bind to the urease enzyme. To the best of our knowledge, all the studies reported in the current manuscript have not been reported in the literature to date.

## 2. Results and Discussion

### 2.1. Chemistry

**Figure 1:**
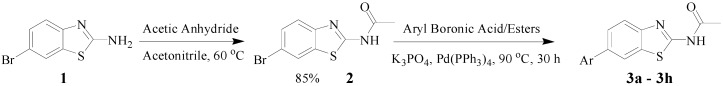
Synthesis of *N*-(6-bromobenzo[d]thiazol-2-yl)acetamide (**2**) and *N*-(6-aryl-benzo[d]thiazol-2-yl)acetamides **3a**–**3h**.

Majo *et al.* reported low to moderate yielding one-step Suzuki cross coupling reactions using various boronic acids/ester with 2-bromobenzothiazole under thermal conditions [18]. We have previously reported Pd(0)-catalyzed reactions of 2-amino-6-bromobenzothiazole with different arylboronic pinacol esters/arylboronic acids using Suzuki cross coupling methodology with moderate yields [19]. We have not been able to achieve better yields as the amino moiety present in the benzothiazole molecule is basic and nucleophilic. In the current study, we have tried to enhance the yield of the synthesized molecules. In order to achieve high yields, the amino group was protected via acylation, which led to substantially enhanced yields of the products **3a**–**3h** (Figure 1) compared to unprotected benzothiazole derivatives as reported in literature [19].

Furthermore, we also optimized other reaction parameters like catalyst loading, solvent, temperature and base used in the reactions producing **3a**–**3h**. Thus, we tried various solvents like toluene, dimethylformamide (DMF) and 1,4-dioxane at different temperatures (80–100 °C). It was noted (in Table 1) that the solvent has an effect on overall reaction yield. 1,4-Dioxane was found to be the best solvent because of its better solvation of the reactants. In our studies, these cross coupling reactions progressed efficiently, even in the presence of known sensitive groups such as CN, to give the desired products in very good yields. Finally after optimization, we investigated the coupling of **2** with different arylboronic pinacol esters/acids in the presence of Pd(PPh_3_)_4_ as catalyst.

Product **3a** was prepared with 80% yield, when **2** was coupled with phenyl boronic acids under the set reaction conditions. The highest yield obtained in this series of reactions (85%) corresponded to product **3b**. Product **3h** was also obtained in excellent yield (83%) with this cross coupling method. It was seen that the product **3c** with an electron withdrawing moiety showed a high yield (81%). Our studies showed that overall the acetylated 2-amino-6-bromobenzothiazole (**2**) gave good yields in these coupling reactions.

### 2.2. Biological Studies

#### 2.2.1. Urease Inhibitory Activity

Urease is a nickel-containing metalloenzyme that catalyzes the hydrolysis of urea to form ammonia and carbamate, which further decomposes to yield a second equivalent of ammonia and carbon dioxide [20]. Bacterial ureases have been reported as an important virulence factor in the development of many harmful clinical conditions for human and animal health. Urease is directly involved in the formation of infectious stones and contributes to pathogenesis [21]. It is the major cause of pathogenesis induced by *Helicobacter pylori*, which plays an important role in peptic ulcers and may lead to stomach cancer. In recent years, a number of compounds have been proposed as urease inhibitors [22,23], which are considered interesting new targets for anti-ulcer drugs and for the treatment of infections caused by urease producing bacteria [24].

**Figure 2:**
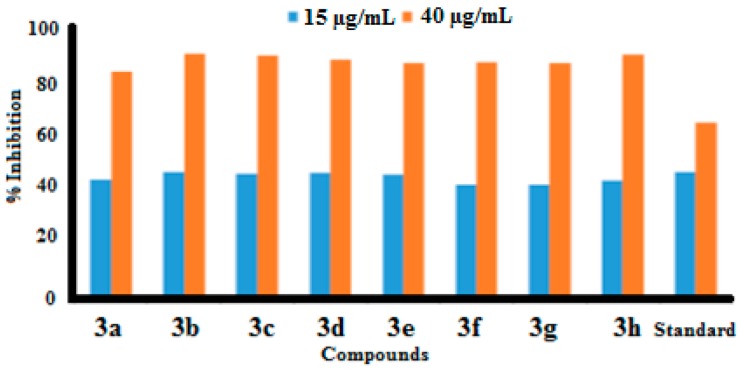
The urease percentage inhibition values at 15 µg/mL and 40 µg/mL.

**Figure 3:**
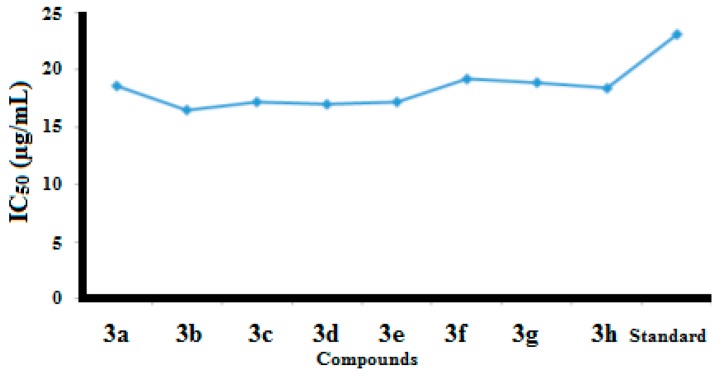
IC_50_ values of anti-urease activity.

In the current study, urease inhibitory activity assays were performed following a previously reported protocol [25]. Thiourea was used as standard in the assay with an IC_50_ value of 23.1 μg/mL. All of the synthesized benzothiazole derivatives were examined for their urease inhibitory activities at concentration of 15 and 40 μg/mL. All of our synthesized compounds exhibit good to excellent urease inhibitory activities (Table 2, Figure 2 and Figure 3).

Urease enzyme has an active binding site and it was believed that the newly synthesized benzothiazole derivatives have the capability to bind to these active sites of urease enzyme. In this way the hydrolysis of enzyme is stopped and activity of enzyme is inhibited. Compound **3b** showed the highest urease inhibition activity (90.51 ± 0.19) with an IC_50_ value of 16.5 μg/mL at 40 μg/mL.

Compounds **3h** and **3c** exhibited excellent urease inhibitory activities (90.07 ± 0.20 and 90.33 ± 0.20 at 40 μg/mL) with IC_50_ values of 17.2 μg/mL and 18.4 μg/mL, respectively. Molecules **3a**, **3d**, **3e**, **3f** and **3g** exhibited good urease inhibition with IC_50_ values of 18.6, 17, 17.2, 19.2 and 18.9 μg/mL, respectively. Notably we observed that the presence of electron donating methyl functional groups produced high urease inhibition. We also noted that different functional groups are responsible for variable antiurease activities of the compounds.

#### 2.2.2. *In Silico* Studies with Urease

To understand the binding of the synthesized compounds with urease *in-silico* studies were performed. Only compounds with IC_50_ values were analyzed for docking studies using the freely available software AutoDock 4.2 and others as described in the Experimental Section. All the compounds were screened at different sites (A & B) of the enzyme. The nickel-containing catalytic site A is the most commonly tested site in the literature [26], while site B is less commonly targeted [20,27]. Our compounds bind more strongly to Site B than to site A. The docking studies results were compared with the experimental results and listed in Table 3 and Table 4. Binding energy, inhibition constant and moldock scores are reported in Table 3, whereas the moldock H-binding energy and number of H-bonding interactions between the test compounds and enzyme are reported in Table 4.

**Figure 4:**
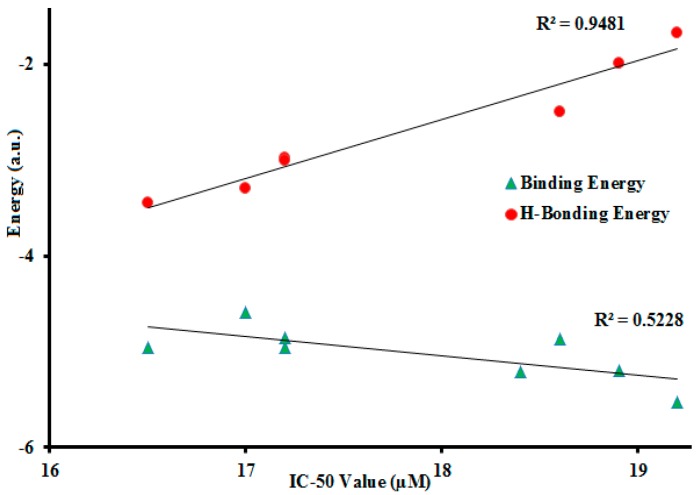
Correlation between docking predicted energies (in arbitrary unit) and *in vitro* IC_50_ values.

The number of H-bonding interactions was estimated from the most stable complex formed between the test compound and the enzyme. We found a linear correlation between the experimental IC_50_ values and the calculated binding energy, as shown in Figure 3. This linear correlation of results is in agreement with the data reported in the literature [26,28]. More interestingly a better correlation was found between IC_50_ values and the moldock H-binding energy (Figure 4).

**Figure 5:**
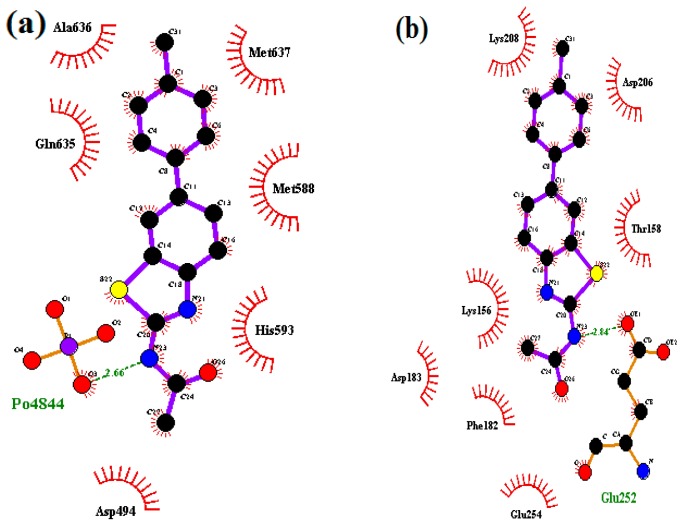
LIGPLOT images of compound **3b** with urease enzyme (**a**) at the catalytic site A; and (**b**) at the catalytic site B.

These results show that the H-bonding is more important, in urease inhibition mechanism, than other factors involved in this biological reaction. Compound **3b**, with the highest *in vitro* activity, is represented here for modeling analysis and its most active conformations are explained in the following paragraphs. As urease is a nickel-dependent enzyme, the active site A of the enzyme shows weak hydrophobic interaction with compound **3b** therefore, a drug with hydrophobic substituents would be able to bind strongly as it would project into the hydrophobic grooves of the enzyme and thus effectively inhibit its activity. The LIGPLOT interaction images show that compound **3b** has a total of seven interactions with enzyme site A (Figure 5a).

The amino acids His593, Met637, Ala636, Gln635, Met588 and Asp494 form hydrophobic interactions with compound **3b**. The hydrophobic interactions favor ligand binding with proteins having metal ions. Furthermore, the study showed hydrogen bonding (2.66 Å, N–H—O type) between compound **3b** and PO_4_ group of the enzyme nickel catalytic site A.

**Figure 6:**
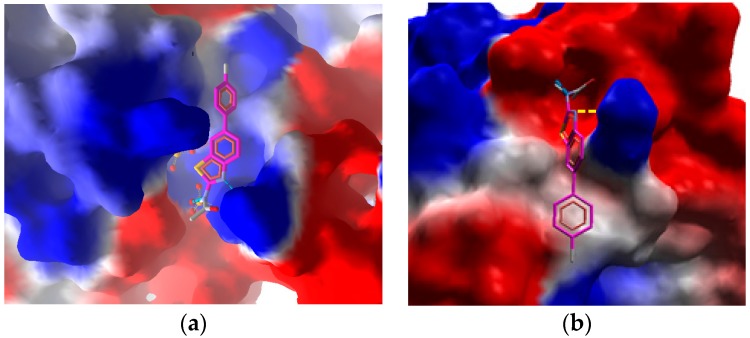
Compound **2c** in the Molegro molecular viewer generated electrostatic surface of urease enzyme (**a**) at the catalytic site A; and (**b**) at the catalytic site B. The yellow dashed line represents the H-bonding.

Figure 6a represents the most interacting conformation of **3b** in the active pocket (site A) of the enzyme at the electrostatic surface. Figure 6 is generated by Molegro Molecular viewer and analyzed by moldock score. The moldock analysis shows that there are two H-bonding interactions in both cases these are also reported in Table 4.

All of the synthesized compounds **3a**–**3h** show better interaction at site B. The LIGPLOT interaction diagram of compound **3b** illustrates that this inhibitor has better interactions with the protein. The LIGPLOT interaction images show that the compound **3b** has a total of eight interactions with enzyme site B (Figure 5b). The amino acids Lys208, Asp206, Thr158, Glu254, Phe182, Lys156 and Asp183 form cationic—π interactions with compound **3b**, while Glu252 interacts via hydrogen bonding (2.84Å, N–H—O type) with compound **3b**. Figure 5b presents the most interacting conformation of compound **3b** in the active pocket of the enzyme (site B). The diagram shows that the enzyme provides enough space for the accommodation of **3b** inside the pocket. The backbone dose sterically favors the **3b** molecule to interact with catalytic site.

**Figure 7:**
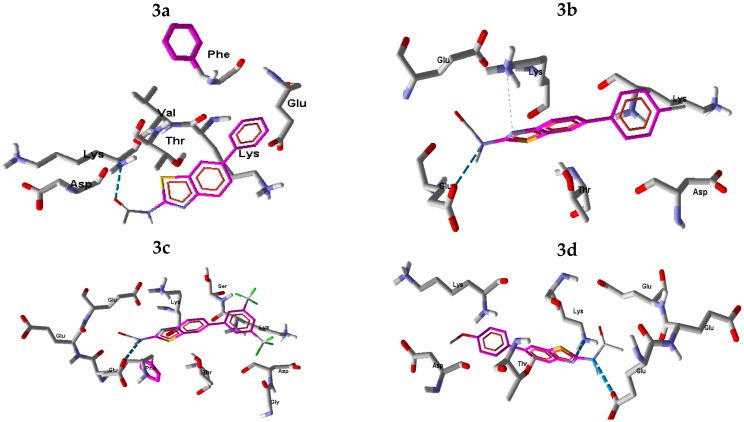

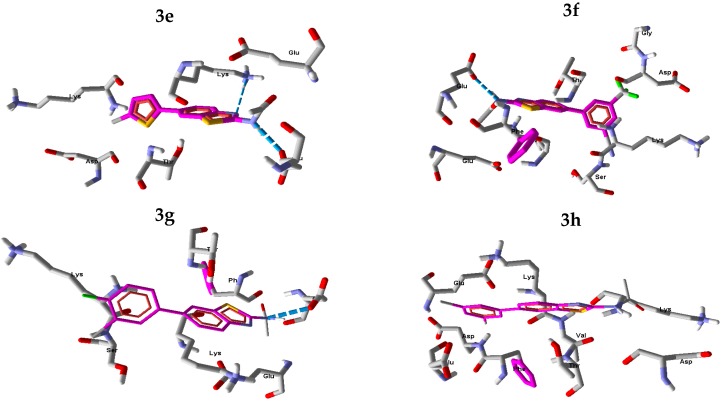
Molegro molecular viewer generated sketches between compounds **3a**–**3h** and the active residue of urease at site B. The blue dashed lines represent the H-bonding.

As shown in Figure 4, a strong correlation with experimental results is found between the IC_50_ values and H-bonding data calculated by moldock in the Molegero docking software at site B of enzyme. In the moldock analysis for **3b** at site A two H-bonding interactions (N–H—O and N–H—N type) were observed between **3b** and PO_4_844, His593, respectively, while two strong H-bonding interactions between **3b** and enzyme residues Glu252 and Lys156 were observed at site B. The H-bonding distances and moldock scores for all compounds, at site B, are listed in Table 4. Figure 7 shows the H-bonding interactions of all of the compounds (except **3h**) with the active residue of urease enzyme at site B. Compound **3h** does not show any H-bonding interaction with the active site residue. In the linear correlation with *in vitro* IC_50_, stronger H-bonding is found for compounds with lower IC_50_ value. All the compounds have H-bonding distances in the 2.696 Å–3.544 Å range and H-bonding energies in the −1.674–−3.45 a.u. range.

#### 2.2.3. Nitric Oxide Scavenging Percentage Assay

**Figure 8:**
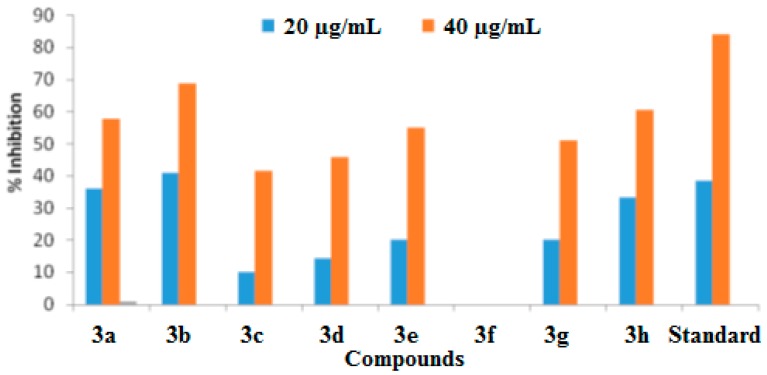
The percentage nitric oxide inhibition at 20 µg/mL and 40 µg/mL.

**Figure 9:**
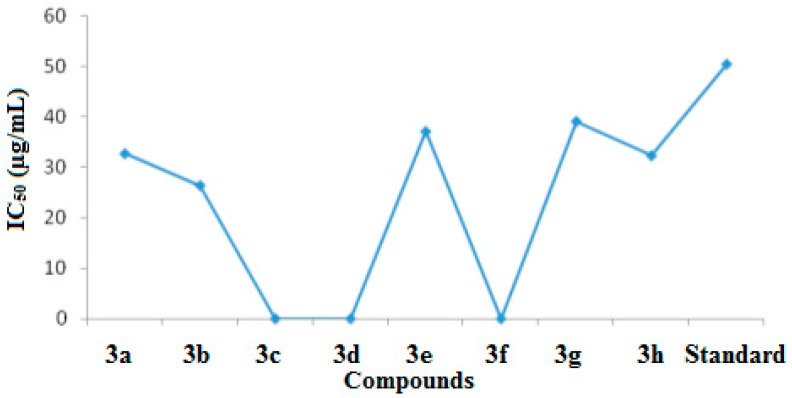
IC_50_ values of nitric oxide scavenging activity.

The literature contains reports on the antioxidant activities of 6-flourobenzothiazole-substituted triazoles using DPPH assays [29]. A survey of the literature showed that benzothiazole molecules along with pyrozoline rings showed the highest antioxidant activities. Having a phenyl ring on the pyrozoline increased the antioxidant activity in the ferric ion reduction and DPPH solution methods [30]. Our newly synthesized *N*-protected benzothiazole derivatives exhibit nitric oxide scavenging activities. Ascorbic acid was used as a standard in the assay with 38.5 ± 0.16 and 84.1 ± 0.12 percent nitric oxide scavenging at 20 μg/mL and at 50 μg/mL with an IC_50_ value of 50.43 μg/mL. Synthesized compounds with their calculated IC_50_ values are listed in Table 5 (Figure 8 and Figure 9).

It was found that acetyl-protected amino group products were more active in the nitric oxide scavenging assay, than the previously reported non-acetylated compounds [19]. Molecules **3a**, **2b**, **3e**, **3g** and **3h** were found to be the most active for nitric oxide scavenging activity, with percentage inhibitions of 57.75 ± 0.12, 69 ± 0.12, 55 ± 0.31, 51.25 ± 0.15 and 60.5 ± 0.1 at 40µg/mL with IC_50_ values of 32.7,26.4, 37.1, 39.1 and 32.3, respectively. Compound **3f**, however, was found to be inactive in the nitric oxide scavenging assay. We are unable to account for this inactivity.

#### 2.2.4. Haemolytic Activity

The haemolytic activity of benzothiazole derivatives has already been reported. A literature survey reveals that amino-substituted derivatives of benzothiazole have high cytotoxicites. Benzothiazole compounds with halogen substitutions show cytotoxicity towards cancer cell lines [31]. The haemolytic activity of the newly synthesized benzothiazole derivatives were studied against Triton X-100 by a reported method [32]. 

**Figure 10:**
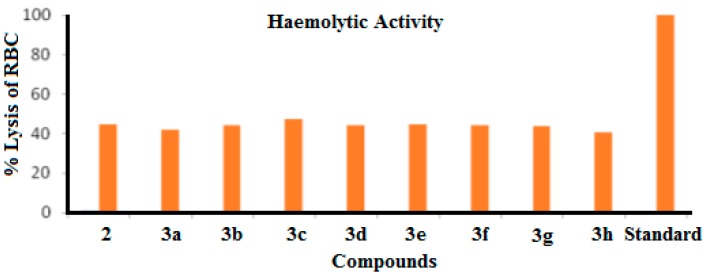
Haemolytic activity of the synthesized compounds.

The newly synthesized benzothiazole derivatives exhibit moderate to high haemolytic activities (see in Table 6, Figure 10). Compound **3c** exhibits the highest haemolytic activity (47.089 ± 0.130). Fluorinated analog **2d** also displayed the highest toxicity among all the tested compounds. The antitumor activity of a compound might be considered as corresponding to the highest haemolytic activity. It was observed that substitution does not markedly affected the haemolytic activity of these newly synthesized *N*-protected benzothiazole derivatives. Compounds **2**, **3b** and **3d**–**3g** showed good haemolytic activities. The lowest haemolytic activity was found for compound **3h**. It was concluded that halogen substitution on *N*-protected benzothiazole molecules promotes haemolytic activity. These compounds have potential to be used as future anticancer agents.

#### 2.2.5. Antibacterial Activity

The synthesized benzothiazole derivatives were examined for their anti-bacterial activity against two Gram positive-bacterial strains (*Baccilus subtiles*, *Staphylococcus aureus*) and four Gram-negative strains (*Escherichia coli*, *Psuedomonas aeruginosa*, *Shigella dysenteriae*, *Salmonella typhae*) at concentrations of 40 and 80 μg/mL (Table 7 and Table 8). It was concluded that the potent antibacterial activities of these compounds might be due to electron withdrawing groups present on the aryl moiety in *N*-protected benzothiazole molecule. Similar observations are also reported by other groups which suggest that the presence of electron releasing and electron withdrawing groups substantially affects the antibacterial activity [33].

The results show that the benzothiazole compounds **3a**–**3h** exhibit higher activities than the standard against some species. Functional group changes in the benzothiazole molecule led to differences in activity. The newly synthesized compounds were found to be inactive against *Baccilus subtilis* and *Staphylococcus aureus*. Only compound **3f** showed activity against *Psuedomonas aeruginosa* with a very small value (7.54 ± 0.6). These newly synthesized compounds do not exhibit considerable antibacterial activity and the highest value (3.51 ± 0.43) was observed for compound **3h** against *Shigella dysenteriae* at concentration of 40 μg/mL. These new benzothiazole molecules showed weak activities against *Salmonella typhae* at 40 μg/mL. It was found that all newly synthesized benzothiazole compounds gave good to very good activity against *E. coli* at 40 μg/mL. Compounds **3c** and **3g**e exhibited very good activities against the *E. coli* strain with values of 49.05 ± 0.32 and 45.93 ± 0.3, respectively. These differences in activities may be attributed to the presence of electron loving atoms/groups on the aryl moiety of these *N*-protected benzothiazole derivatives.

The synthesized compounds were also checked for antibacterial activities at 80 μg/mL and compared against ampicillin. It was shown that these compounds showed moderate activities against *Bacillus subtiles* with the highest value (12.66 ± 0.531) corresponding to compound **3e**. The authors concluded that these benzothiazole derivatives showed non-significant activity against *Staphylococcus aureus*. In addition, these compounds were to be found active against *Shigella dysenteriae* and *Salmonella typhae* at 80 μg/mL. These compounds displayed very good activity against *E. coli* at 80 μg/mL. The benzothiazole derivatives were discovered to be the most potent against the *E. coli* strain. Compounds **3a**, **3b** and **3g** proved to be the most potent at the concentration of 80 μg/mL with the highest antibacterial activities with values of 57.97 ± 0.25, 57.84 ± 0.25 and 56.13 ± 0.32, respectively. The results of this study revealed that electron withdrawing group substitution on the aryl moiety on the benzothiazole molecule enhanced the antimicrobial activity of the compounds.

## 3. Experimental Section

### 3.1. General Information

All reagents and chemicals were brought from Alfa-Aesar Chemical Co. (Ward Hill, MA, USA) and Sigma-Aldrich Chemical Co. (St. Louis, MO, USA). Solvents CDCl_3_ and CD_3_OD were used for ^13^C-NMR and ^1^H-NMR spectra on an Aspect AM-400 instrument at 400/100 MHz (Bruker, Billerica, MA, USA). The coupling constant was determined in Hz and chemical shift in δ in ppm. A JMS-HX-110 spectrometer (JEOL, Peabody, MA, USA) was used for EI-MS spectra. Melting points of benzothiazole compounds were measured on a B-540 melting point apparatus (Büchi, New Castle, DE, USA). Column chromatography with silica gel (mesh size 70 to 230 and 230 to 400) was used for compound purification. TLC (silica gel 60 PF 254 cards, Merck, Kenilworth, NJ, USA) was used for reaction monitoring. Plates were visualized using a UV lamp (254 to 365 nm) (Spectronics Corporation, Westbury, NY, USA).

### 3.2. Procedure for the Preparation of N-(6-arylbenzo[d]thiazole-2-yl)acetamides ***3a**–**3h***

The preparation of products **3a**–**3h** was carried out under a nitrogen atmosphere. Compound **2** (synthesized by literature reported method [19]) (2.183 mmol) and 5 mol % Pd(PPh_3_)_4_ in 1,4-dioxane (20 mL) was mixed and stirred for 30 min. After 30 min K_3_PO_4_ (4.366 mmol), aryl boronic pinacol esters/aryl boronic acids (2.401 mmol), and H_2_O (1.5 mL) was added under inert atmospheric conditions. The mixture was stirred for 30 h at 95 °C and cooled down to room temperature. Ethyl acetate was used for work up and the organic layer was separated and dried under vacuum. For purification purposes, column chromatography was done. The desired product was obtained by using ethylacetate and *n*-hexane (20% and 80%respectively) as eluents. The desired products were characterized by various spectroscopic techniques [34].

*N-(6-Phenylbenzo[d]thiazol-2-yl)acetamide*(**3a**).m.p. 203–205 °C; ^1^H-NMR (CDCl_3_ + CD_3_OD) δ 9.46 (s, 1H), 7.92 (s, 1H), 7.67–7.50 (m, 4H), 7.46–7.31 (m, 3H), 2.31 (s, 3H); ^13^C-NMR (CDCl_3_ + CD_3_OD) δ 168.3, 159.2, 151.9, 145.6, 132.4, 130.1, 129.8 (2C), 128.4, 128.4 (2C), 124.2, 121.7, 116.8, 23.7; EIMS (*m*/*z* + ion mode): 269.32 [M + H^+^] 269.08; Anal Calcd for C_15_H_12_N_2_OS: C, 67.13; H, 4.53; N, 10.43; found C, 67.23; H, 4.54; N, 10.37.

*N-(6-(p-Tolyl)benzo[d]thiazol-2-yl)acetamide*(**3b*)***.m.p. 205–207 °C; ^1^H-NMR (CDCl_3_ + CD_3_OD) δ 9.33 (s, 1H), 8.11 (d, *J =* 10 Hz, 3H), 7.61 (d, *J =* 10.4 Hz, 1H), 7.31–7.18 (m, 3H), 2.41 (s, 3H), 2.35 (s, 3H); ^13^C-NMR (CDCl_3_ + CD_3_OD) δ 168.2, 159.3, 148.4, 146.8, 133.1, 131.3, 129.6 (2C), 128.4 (2C), 126.1, 124.4, 121.2, 116.8, 22.9, 21.6; EIMS (*m*/*z* + ion mode): [M + H^+^] = 283.08; [M − COCH_3_]^−^ = 241.01. Anal Calcd for C_16_H_14_N_2_OS: C, 68.07; H, 5.01; N, 9.93 found C, 68.12; H, 5.04; N, 9.81.

*N-(6-(3,5-bis(Trifluoromethyl)phenyl)benzo[d]thiazol-2-yl)acetamide* (**3c**). m.p. 203–205 °C; ^1^H-NMR (CDCl_3_ + CD_3_OD) δ 9.41 (s, 1H), 7.92 (s, 1H), 7.66–7.61 (m, 2H), 7.55–7.43 (m, 3H), 2.27 (s, 3H); ^13^C-NMR (CDCl_3_ + CD_3_OD) δ 168.7, 159.4, 153.7, 146.3, 137.1, 132.1 (2C), 129.3, 128.4 (2C), 124.1 (2C), 123.3, 121.4, 120.12, 118.6, 25.2; EIMS (*m*/*z* + ion mode): 405.32, [M + H]^+^; 405.16. Anal Calcd for C_17_H_10_F_6_N_2_OS: C, 50.51; H, 2.48; N, 6.92 found C, 50.44; H, 2.52; N, 6.82.

*N-(6-(4-Methoxyphenyl)benzo[d]thiazol-2-yl)acetamide* (**3d**). m.p.: 218–220 °C; ^1^H-NMR (CDCl_3_ + CD_3_OD) δ 9.41 (s, 1H), 8.01 (s, 1H), 7.66 (d, *J* = 8, 2H), 7.57–7.47 (m, 2H), 7.08 (d, *J* = 8, 2H), 3.57 (s, 3H), 1.91 (s, 3H). ^13^C-NMR (CDCl_3_ + CD_3_OD) δ 168.2, 158.8, 151.3, 149.5, 145.3, 132.2, 131.8 (2C), 129.5, 123.7, 121.3, 116.6, 115.5 (2C), 49.2, 22.5; EIMS (*m*/*z* − ion mode): [M − H]^−^ = 297.25; [M − COCH_3_]^−^ = 255.34; [M − OCH_3_ and COCH_3_]^−^ = 227.01; Anal Calcd for C_16_H_14_N_2_O_2_S_2_: C, 64.42; H, 4.73; N, 9.38 found C, 64.51; H, 4.71; N, 9.42.

*N-(6-(5-Methylthiophen-2-yl)benzo[d]thiazol-2-yl)acetamide* (**3e**). m.p.: 192–194 °C; ^1^H-NMR (CDCl_3_ + CD_3_OD) δ 9.41 (s, 1H), 7.90 (s, 1H), 7.67–7.35 (m, 4H), 2.34 (s, 3H), 2.11 (s, 3H); ^13^C-NMR (CDCl_3_ + CD_3_OD) δ 166.2, 159.6, 148.7, 145.2, 132.4, 132.3, 129.5, 128.7, 128.3, 123.6, 121.8, 116.8, 22.7, 14.03; EIMS (*m*/*z* − ion mode): [M − H]^−^ = 286.93; [M − COCH_3_]^−^ = 224.93; [M − CH_3_]^−^ = 271.02; Anal Calcd for C_14_H_12_N_2_OS_2_: C, 58.32; H, 4.20; N, 9.72 found C, 58.41; H, 4.12; N, 9.79.

*N-(6-(3-Cyano-5-(trifluoromethyl)phenyl)benzo[d]thiazol-2-yl)acetamide* (**3f**). m.p.: 212–213 °C; ^1^H-NMR (CDCl_3_ + CD_3_OD) δ 9.48 (s, 1H),7.92 (d, *J =* 1.2, 1H), 7.72–7.41 (m, 4H), 7.36 (s, 1H), 2.27 (s, 3H); ^13^C-NMR (CDCl_3_ + CD_3_OD) δ 167.7, 158.8, 148.7, 146.4, 132.3, 132.0, 132.0, 131.8, 129.6, 128.4, 124.2, 123.3, 121.8, 120.3, 117.7, 114.8, 22.5; EIMS (*m*/*z* − ion mode): 360.33 [M − H]^−^ 360.01; Anal Calcd for C_17_H_11_F_3_N_3_OS: C, 56.52; H, 2.78; N, 11.62 found C, 56.60; H, 2.77; N, 11.57.

*N-(6-(3-Chloro-4-fluorophenyl)benzo[d]thiazol-2-yl)acetamide* (**3g**). m.p.: 166–167 °C; ^1^H-NMR (CDCl_3_ + CD_3_OD) δ 9.32 (s, 1H), 8.11 (d, *J =* 10 Hz, 1H), 7.90 (s, 1H), 7.80–7.41 (m, 4H), 2.30 (s, 3H); ^13^C-NMR (CDCl_3_ + CD_3_OD) δ 168.5, 156.3, 151.2, 147.8, 137.3, 135.1, 131.0, 129.7, 129.6, 124,6, 124.0, 121.6, 117.5, 116.3, 22.5; EIMS (*m/z* + ion mode): [M + H]^+^ = 321.16; [M − Cl and F-benzene and COCH_3_]^+^ = 150.07; Anal Calcd for C_15_H_12_ClFN_2_OS: C, 56.16; H, 3.13; N, 8.72 found C, 56.30; H, 3.15; N, 8.76.

*N-(6-(3,5-Dimethylphenyl)benzo[d]thiazol-2-yl)acetamide* (**3h**). m.p. 205–207 °C; ^1^H-NMR (acetone-*d*_6_) δ 9.41 (s, 1H),8.14 (d, *J =* 2 Hz, 1H), 7.63 (d, *J =* 1.5, 1H), 7.72–7.53 (m, 4H), 2.31 (s, 6H), 2.28 (s, 3H); ^13^C-NMR (acetone-*d*_6_) δ 167.2, 158.0, 147.7, 140.3 (2C), 133.0, 129.9, 129.5, 128.3, 127.4 (2C), 123.7, 121.3, 119.2, 23.1, 22.2 (2C); EIMS (*m*/*z* − ion mode): [M − H]^−^ = 294.91; [M − COCH_3_ − 2OCH_3_ and benzene]^−^ = 149.24; [M − 2OCH_3_ and benzene]^−^ = 190.6; Anal Calcd for C_17_H_16_N_2_OS: C, 68.88; H, 5.43; N, 9.44 found C, 68.83; H, 5.46; N, 9.34.

### 3.3. Procedure for Urease Inhibition Activity

Urease inhibitory assay of newly synthesized compounds **3a**–**3h** were determined as follows: Enzyme (1 unit) in phosphate buffer (200 µL, pH 7) was combined with a particular stock solution (20 µL, a test compound or thiourea) and phosphate buffer (230 µL). The solution was incubated for 5 min at 25 °C. After incubation period 400 µL of urea stock solution (20 mM) was added to the solution. Calibration solution was synthesized without urea solution. For the action of urease, test tubes were incubated for 10 min at 40 °C. The solution of phenol hypochlorite reagent (1150 µL) was added. These tubes were incubated for 25 min at 56 °C. Absorbance of the blue colored compound was noted at 625 nm after 5 min of cooling. Then percentage urease inhibition was determined. While EZ-fit kinetic data base was used to obtain IC_50_ values [25,35].

### 3.4. Molecular Docking Study

The PDB structure of 3LA4 was retrieved for docking purposes as a complex co-crystallized with inhibitor 2-amino-3-(2-(2-hydroxyethyl)disulfanyl)propan-1-ol and (5-amino-6-hydroxyhexyl)carbamic acid at the nickel-containing catalytic site [36]. Then, the amino acid chain was retained and the water molecules and co-crystallized ligands were removed and subsequently the missing atom types were repaired using Modeller 9.11 (University of California San Francisco, San Francisco, CA, USA). Afterwards, the polar hydrogen was added to the receptor and the resulting protein was subjected to minimization using OPLS 2005 force field. The prepared protein was saved in pdbqt format using Autodock Tools 1.5.4 [21,37,38,39]. The ligand coordinates were generated using MarvineSketch 5.8.3, 2012 (ChemAxon LLC, Cambridge, MA, USA) [40], which was converted to 3D structure using Openbabel version (2.3.1). Finally the pdbqt formats (The input format of docking software) of the ligands were prepared with Autodock Tools 1.5.4 using default parameters. AutodockVina ver. 1.1.1 (The Scripps Research Institute, La Jolla CA, USA) was used for docking calculations with default parameters except for exhaustiveness that was set to 80. For all the docking calculations, a grid box size of 40 × 40 × 40, centered at the geometrical center of co-crystallized ligands was used. Co-crystalized ligands were attached at two different sites, one near the nickel catalytic site (A) and the other site where the inhibitor 2-amino-3-(2-(2-hydroxyethyl)disulfanyl)propan-1-ol was attached (Site B) [20]. The coordinates x, y, z for the center of grid box were (Site A) −39.86, −45.06, 72.52 and (Site B) −75.03, 20.84, 81.83 respectively. To validate our docking procedure, the co-crystallized ligands were re-docked into their respective site of the enzyme and the reasonable RMSD value of 1.947 A was obtained. Finally, the conformations with the most favorable free energy of binding were selected for analyzing the interactions between urease and its inhibitor. All of the 3D models are generated using the Molegro Molecular Viewer 2.5 (CLC bio company, Aarhus N, Denmark) [41] and LigPlot + (The European Bioinformatics Institute, Hinxton, Cambridge, UK) [42] software.

### 3.5. Nitric Oxide Scavenging Activity

The activity of newly synthesized benzothiazole derivatives was determined using the Garrat method. Griess reported the Garrat method which is followed by a diazotization reaction [43]. Under acidic conditions, this method utilizes sodium nitroprusside as the source of nitric oxide, sulfanilamide and *N*-1-naphthylethylenediamine dihydrochloride to detect NO_2_^−^, produced at the expense of nitric oxide. A known amount of tested compounds **3a**–**3h** was dissolved in sodium nitroprusside solution (20 mM, 100 µL) and then the volume was made up to 1000 µL with phosphate buffer (200 mM, pH 7.4). This solution was incubated for 2 h at 37 °C and Griess reagent (100 µL) was added. This solution was stored for 20 min at room temperature. At 528 nm, optical density of this colored solution was observed. For positive control, ascorbic acid was used. Negative control was used to form the standard curve [43].

### 3.6. Haemolytic Activity

Haemolytic activity of newly synthesized benzothiazole derivatives **3a**–**3h** was determined using a reported method [44]. Solutions of compounds were prepared at concentrations of 1 mg/mL in 10% DMSO with 90% water Heparinized human fresh blood (3 mL) was used that was homogeneously mixed and added into a 15 mL sterile Falcon tube. It was centrifuged for 5 min and the supernatant was removed. Chilled sterile isotonic phosphate buffer saline solution (5 mL, 7.4 pH) at 4 °C was used three times. Washed red blood cells were suspended in chilled RBS (20 mL). A haemacytometer was used for counting erythrocytes. For each assay 7.068 × 10^8^ red blood cells per mL count were maintained and then diluted blood cells (180 µL) were added to the test compound (20 µL) and suspended in Eppendorf tubes. It was incubated for 35 min at 37 °C then the tubes were kept in an ice bath for 5 min and centrifuged again for 5 min. After centrifugation, the obtained supernatant was collected carefully and diluted with 900 µL of chilled PBS. All these tubes were kept in ice bath and solution (200 µL) was added into 96 well plates from each Eppendrof tube. For each essay, Triton X-100 (0.1%) was taken as positive control. For negative control, phosphate buffer was used. A microplate reader was used for determining the absorbance at 576 nm [32].

### 3.7. Antibacterial Activity

These newly benzothiazole derivatives were tested for their antibacterial activities against two Gram positive strains (*Baccilus subtilis, Staphylococcus aureus)* and four Gram negative strains (*Escherichia coli, Psuedomonas aeruginosa, Shigella dysenteriae* and *Salmonella typhae*) using a reported protocol [45]. Streptomycin was used as positive control. The 96 well plate method was optimized for measuring the antibacterial activities of these compounds. In each well, sterilized broth (175 µL) was added and inoculated with glycerol stock (5 µL) of a specific bacterial strain. The initial absorbance was observed between 0.12–0.19. The bacteria were allowed to grow in an incubator overnight. After a certain waiting time (12 h), test sample (20 µL) was added to the pre-determined wells. Concentration of test sample was 20 µg/well. Total volume was 200 µL in each well. These plates were incubated for 16–24 h at 37 °C. Absorbance was observed at 630 nm by using an ELISA plate reader. The difference in absorbance values were observed and were used as an index of bacterial growth. The following formula was used to calculate percentage inhibition:

Percentage Inhibition = (O.D of + ve control − O.D of sample × 100)/O.D of +ve control
(1)

## 4. Conclusions

This study reports C-C coupling reactions of **2** with various arylboronic pinacol ester/aryl boronic acids using palladium catalyst. These new products **3a**–**3h** were prepared in moderate to good yields. These Suzuki coupling benzothiazole derivatives were checked for their biological (urease inhibitory, nitric oxide scavenging, haemolytic and antibacterial) activities. The urease inhibition results showed that product **3b** was an excellent urease inhibitor. Products with electron releasing groups on the aryl moiety of the benzothiazole molecule showed the highest inhibition of urease activity. Molecular docking studies of the urease inhibitory activity showed that the H-bonding ability present in these *N*-protected benzothiazoles prevents the catalytic activity of the enzyme. Nitric oxide scavenging assays were done for these compounds. Compound **3b** also exhibited highest nitric oxide scavenging activity. All newly synthesized compounds showed haemolytic activity. It was found that electron withdrawing substitution on the aryl produced the highest haemolytic activity. The newly synthesized benzothiazole derivatives **3a**–**3h** showed excellent antibacterial activities against *E. coli*.

## Figures and Tables

**Table 1:** Synthesis of *N*-(6-arylbenzo[d]thiazol-2-yl)acetamides **3a**–**3h.**

Entry	Arylboronic Pinacol Ester/Arylboronic Acid	Product	H_2_O/Solvent (1:4)	Yields %
1	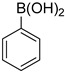	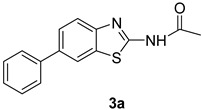	Toluene 1,4-Dioxane	75 80
2	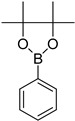	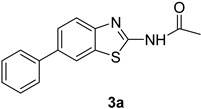	1,4-Dioxane	77
3	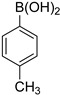	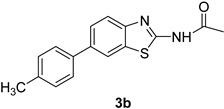	1,4-Dioxane	85
4	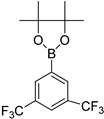	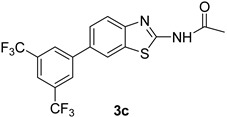	1,4-Dioxane	81
5	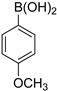	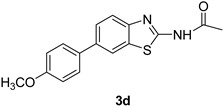	1,4-Dioxane	79
6	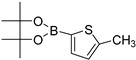	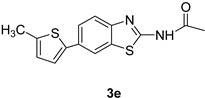	1,4-Dioxane	75
7	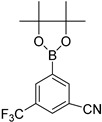	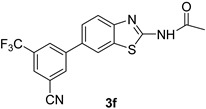	1,4-Dioxane	77
8	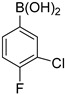	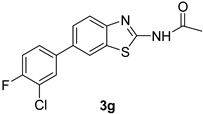	1,4-Dioxane	79
9	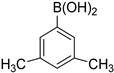	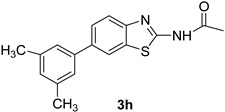	1,4-Dioxane	83

**Table 2:** Antiurease activity of *N*-(6-arylbenzo[d]thiazole-2-yl)acetamides **3a**–**3h** at 15µg/mL and 40 µg/mL.

Compound	% Inhibition at 15 µg/mL	% Inhibition at 40 µg/mL	IC_50_ (µg/mL)
**3a**	44 ± 0.12	83.9 ± 0.12	18.6
**3b**	47 ± 0.11	90.51 ± 0.19	16.5
**3c**	46.09 ± 0.10	90.07 ± 0.20	17.2
**3d**	46.5 ± 0.15	88.5 ± 0.24	17
**3e**	46 ± 0.12	87 ± 0.26	17.2
**3f**	42 ± 0.12	87.5 ± 0.26	19.2
**3g**	42 ± 0.14	87 ± 0.26	18.9
**3h**	43.59 ± 0.13	90.33 ± 0.20	18.4
Standard	47 ± 0.31	65 ± 0.01	23.1

Each values is mean ± Standard deviation of three parallel measurements.

**Table 3:** Experimental and docking comparative data.

Compound	* IC_50_ (μg/mL)	Inhibition Constant	Binding Energy	Moldock Score
**3a**	18.6	267.93	−4.87	−83.39
**3b**	16.5	232.29	−4.96	−81.68
**3c**	17.2	232.56	−4.96	−68.81
**3d**	17.0	434.76	−4.59	−69.20
**3e**	17.2	278.10	−4.85	−87.00
**3f**	19.2	90.570	−5.52	−87.90
**3g**	18.9	153.46	−5.20	−79.91
**3h**	18.4	150.67	−5.21	−80.11

* Experimentally measured *in vitro.*

**Table 4:** H-bonding parameters calculated by Mol-Dock Molegero Molecular Viewer 2.5.

Compound	Number of H-bonds	H-Bonding Type (K--H--L) *	H-Bond Distance (K–L) (Å)	IC_50_ (μg/mL)	H-Binding Energy
**3a**	1	O—H–N	2.957	18.6	−2.50
**3b**	2	N—H–N N–H—O	2.837 3.544	16.5	−3.45
**3c**	1	N–H—O	2.782	17.2	−2.98
**3d**	2	N–H—O N—H–N	3.023 3.23	17.0	−3.30
**3e**	2	N–H—O N—H–N	2.906 3.388	17.2	−3.01
**3f**	1	N–H—O	2.739	19.2	−1.67
**3g**	1	N–H—O	2.696	18.9	−1.99
**3h**	zero	--	--	18.4	--

* K atom from the compound and L atom from the protein residue.

**Table 5:** Nitric oxide scavenging activity of *N*-(6-arylbenzo[d]thiazole-2-yl)acetamides **3a**–**3h** at 20 µg/mL and 40 µg/mL.

Compound	% Activity at 20 µg/mL	% Activity at 40 µg/mL	IC_50_ (µg/mL)
**3a**	36.25 ± 0.12	57.75 ± 0.12	32.7
**3b**	41 ± 0.11	69 ± 0.12	26.4
**3c**	10 ± 0.18	41.75 ± 0.20	NC
**3d**	14.25 ± 0.17	46 ± 0.2	NC
**3e**	20.25 ± 0.15	55 ± 0.31	37.1
**3f**	0	0	NC
**3g**	20.25 ± 0.15	51.25 ± 0.15	39.1
**3h**	33.5 ± 0.13	60.5 ± 0.1	32.3
Standard	38.5 ± 0.16	84.1 ± 0.12	50.43

Each value is mean ± Standard deviation of three parallel measurements. NC stands for not calculated due to less activity.

**Table 6:** Haemolytic activity of newly synthesized *N*-(6-bromobenzo[d]thiazol-2-yl)acetamide (**2**) and *N*-(6-arylbenzo[d]thiazole-2-yl)acetamides (**3a**–**3h**).

Entry	% lysis of RBC	Entry	% lysis of RBC
**2**	44.628 ± 0.369	**3e**	44.425 ± 0.181
**3a**	42.123 ± 0.479	**3f**	44.063 ± 0.314
**3b**	44.179 ± 0.157	**3g**	43.614 ± 0.157
**3c**	47.089 ± 0.130	**3h**	40.661 ± 0.216
**3d**	44.078 ± 0.279		
Standard	99.78 ± 0.912		

Each value is mean ± Standard deviation of three parallel measurements.

**Table 7:** Antibacterial activity (40 μg/mL) of *N*-(6-arylbenzo[d]thiazole-2-yl)acetamides **2b**–**2i**.

Entry	% Activity at 40 μg/mL					
	*B. subtilis*	*S. aureus*	*P. aeruginosa*	*S. dysenteriae*	*S. typhae*	*E. coli*
**3a**	-	-	-	0.94 ± 0.45	-	37.52 ± 0.38
**3b**	-	-	-	-	-	34.04 ± 0.40
**3c**	-	-	-	0 ± 0.45	6.0 ± 0.47	49.05 ± 0.32
**3d**	-	-	-	2.92 ± 0.44	-	42.10 ± 0.36
**3e**	-	-	-	-	-	37.82 ± 0.38
**3f**	-	-	7.54 ± 0.6	2.59 ± 0.44	1.2 ± 0.50	33.53 ± 0.41
**3g**	-	-	-	1.45 ± 0.44	-	45.93 ± 0.3
**3h**	-	-	-	3.51 ± o.43	-	22.89 ± 0.47
**Ampicillin**	23 ± 0.1	29 ± 0.61	25 ± 0.12	35 ± 0.32	29 ± 0.61	19 ± 0.31

Each value is mean ± standard deviation of three parallel measurements.

**Table 8:** Antibacterial activity (80 μg/mL) of *N*-(6-arylbenzo[d]thiazole-2-yl)acetamides **2b**–**2i**.

Entry	% Activity at 80 μg/mL					
	*B. subtilis*	*S. aureus*	*P. aeruginosa*	*S. dysenteriae*	*S. typhae*	*E. coli*
**3a**	6.08 ± 0.571	15.25 ± 0.5	-	8.47 ± 0.44	18.5 ± 0.58	57.97 ± 0.25
**3b**	-	-	-	6.57 ± 0.45	17.7 ± 0.5	56.13 ± 0.32
**3c**	-	-	-	-	26.2 ± 0.53	50.49 ± 0.30
**3d**	7.31 ± 0.5635	-	-	5.92 ± 0.46	28.86 ± 0.5	51 ± 0.30
**3e**	12.66 ± 0.531	3.75 ± 0.65	-	-	19.2 ± 0.5	50.8 ± 0.30
**3f**	4.27 ± 0.582	8.89 ± 0.5	2.96 ± 0.6	10.93 ± 0.43	25 ± 0.54	55 ± 0.32
**3g**	-	-	-	11.51 ± 0.42	21.05 ± 0.5	57.84 ± 0.25
**3h**	5.67 ± 0.5735	-	-	7.48 ± 0.45	17.43 ± 0.5	53.96 ± 0.40
**Ampicillin**	50.5 ± 0.31	52.9 ± 0.29	52 ± 0.26	56 ± 0.26	42.9 ± 0.29	45.9 ± 0.21

Each value is mean ± standard deviation of three parallel measurements.
